# Phylogenetic Analysis Reveals the Global Migration of Seasonal Influenza A Viruses

**DOI:** 10.1371/journal.ppat.0030131

**Published:** 2007-09-14

**Authors:** Martha I Nelson, Lone Simonsen, Cecile Viboud, Mark A Miller, Edward C Holmes

**Affiliations:** Center for Infectious Disease Dynamics, The Pennsylvania State University, University Park, Pennsylvania, United States of America; Department of Biology, The Pennsylvania State University, University Park, Pennsylvania, United States of America; National Institute of Allergy and Infectious Diseases, National Institutes of Health, Bethesda, Maryland, United States of America; Fogarty International Center, National Institutes of Health, Bethesda, Maryland, United States of America; Emory University, United States of America

## Abstract

The winter seasonality of influenza A virus in temperate climates is one of the most widely recognized, yet least understood, epidemiological patterns in infectious disease. Central to understanding what drives the seasonal emergence of this important human pathogen is determining what becomes of the virus during the non-epidemic summer months. Herein, we take a step towards elucidating the seasonal emergence of influenza virus by determining the evolutionary relationship between populations of influenza A virus sampled from opposite hemispheres. We conducted a phylogenetic analysis of 487 complete genomes of human influenza A/H3N2 viruses collected between 1999 and 2005 from Australia and New Zealand in the southern hemisphere, and a representative sub-sample of viral genome sequences from 413 isolates collected in New York state, United States, representing the northern hemisphere. We show that even in areas as relatively geographically isolated as New Zealand's South Island and Western Australia, global viral migration contributes significantly to the seasonal emergence of influenza A epidemics, and that this migration has no clear directional pattern. These observations run counter to suggestions that local epidemics are triggered by the climate-driven reactivation of influenza viruses that remain latent within hosts between seasons or transmit at low efficiency between seasons. However, a complete understanding of the seasonal movements of influenza A virus will require greatly expanded global surveillance, particularly of tropical regions where the virus circulates year-round, and during non-epidemic periods in temperate climate areas.

## Author Summary

The winter seasonality of influenza A virus in temperate climates is one of the most puzzling epidemiological patterns in infectious disease. To help resolve the issue of influenza seasonality, we studied, using viral genome sequence data, the patterns of global migration of influenza A virus, particularly between the northern and southern hemispheres. A phylogenetic analysis of approximately 900 complete genomes of the H3N2 subtype of human influenza A virus sampled from New Zealand and Australia (southern hemisphere), and New York state, United States (northern hemisphere), revealed that cross-hemisphere migration frequently occurs in both directions and involves multiple viral strains. Such global viral traffic therefore contributes significantly to the introduction of new influenza epidemics in both northern and southern hemispheres. These results also show that influenza A virus migrates afar during non-epidemic periods, rather than persisting locally at low levels during the influenza “off-season”. However, although this represents the largest and first bihemisphere study of its kind to our knowledge, the results highlight the need for sampling from tropical regions and during non-epidemic periods in temperate areas. Studies of this kind are critical to fully understand the geographical dispersal of influenza A virus and the role of climate in triggering seasonal epidemics.

## Introduction

Influenza A virus is able to persistently re-infect human populations by continually evading host immunity through the rapid evolution of surface antigens (“antigenic drift”) [1]. Influenza virus epidemics strike temperate latitudes of the world each winter, from November to March in the northern hemisphere and from May to September in the southern hemisphere [2]. In the United States alone, these influenza epidemics are associated with an annual average of 36,000 human deaths [3] and 226,000 hospitalizations [4]; globally, the virus is associated with as many as half a million annual deaths [5]. While rapid antigenic change is a hallmark of influenza evolution, recent studies have failed to detect antigenic drift over an epidemic season, suggesting that important evolutionary processes may occur during non-epidemic periods, either locally or perhaps elsewhere [6–8]. However, surveillance during non-epidemic periods is not conducted routinely by the network of World Health Organization influenza reference centers [9] and, consequently, little is known about how and where the virus persists in the human population in between winter epidemics at low levels. A key question is therefore whether the virus remains locally within its host population in between epidemics, perhaps persisting within hosts in a latent state [10], or whether the virus migrates afar to other reservoirs, such as the tropics, and is later reintroduced.

Although influenza virus has long been regarded a “cold-weather” pathogen due to its marked winter epidemics in temperate zones, recent studies show that tropical regions experience significant year-round influenza virus activity [11]. In theory, such a “tropical belt” could serve as a year-round reservoir that harbors endemic populations of influenza virus that seasonally reintroduce viral isolates into temperate zones to trigger new epidemics [12,13]. Whereas population crashes at the end of seasonal epidemics create severe evolutionary bottlenecks that limit genetic diversity, tropical zones may function as permanent mixing pools for viruses from around the world. Historically, Southeast Asia has been considered a potential epicenter for emergence of pandemic viruses due to the proximity with which humans live with their domestic animals [14]. However, abundant data from these regions is currently unavailable, so the origins of influenza pandemics and epidemics remain unclear.

Given the ease and speed with which the influenza virus is thought to spread between humans, it is generally accepted that global chains of direct person-to-person transmission are sufficient to maintain the influenza virus in the human population [15]. However, a complete understanding of how the influenza virus transmits between humans is lacking [16], and whether human-to-human spread alone accounts for the seasonal emergence of epidemics has been questioned [17]. The simultaneous appearance of influenza outbreaks separated by large longitudinal distances, as well as sporadic influenza cases during summer months, suggests that the virus may instead already be “seeded” and somehow reactivated by environmental stimuli. Thus, the alternating pattern of northern and southern hemisphere bi-epidemics could, in principle, also result from opposite climatic forces independently reactivating viral activity in these two hemispheres at alternating six-month intervals. Hence, instead of continually migrating across the equator, separate viral populations could persist locally in an asymptomatic latent state over the summer months until climatic stimuli sufficiently increase host susceptibility and/or viral transmissibility to induce another epidemic. However, hypotheses of how climatic change may directly or indirectly influence viral activity and/or host susceptibility remain largely untested [18,19].

**Figure 1:**
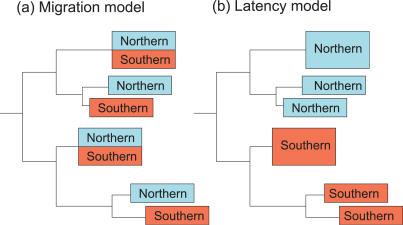
Models for the Global Evolution of Influenza Virus Two phylogenetic hypotheses depicting the evolution of A/H3N2 influenza virus by (A) global migration, in which isolates from adjacent seasons in the northern and southern hemispheres are interspersed topologically, or by (B) reactivation of latent virus, in which isolates from the northern hemisphere give rise, in situ, to isolates that circulate in the same locality the next season, and thus are topologically linked (likewise for southern hemisphere viruses).

Crucially, some theories for influenza seasonality produce testable phylogenetic hypotheses. On one hand, if influenza A virus persists locally over the summer in a latent state, then isolates sampled over multiple seasons from a single locality would cluster together on a phylogenetic tree, separate from isolates from other geographic regions (Figure 1). Alternatively, if the virus did not evolve in situ between epidemic seasons, but rather traveled globally between epidemics, then the resulting phylogeny would show extensive intermixing of isolates from different localities. To determine whether influenza virus migrates away between the northern and southern hemispheres during non-epidemic summer months, or remains there latently, we conducted an extensive phylogenetic analysis of 399 whole-genome A/H3N2 influenza A viruses sampled from New Zealand (most commonly Canterbury, South Island) from 2000 to 2005 (six seasons), 88 viral genome sequences from Australia (most commonly Western Australia) from 1999 to 2005 (seven seasons), along with a carefully selected sub-sample of 52 isolates that are representative of the clades present in a larger sample of 413 viruses from New York state, United States, collected between 1998 and 2005 and analyzed previously [7]. Given the relative geographic isolation and low population densities of New Zealand's South Island and Western Australia, as well as sampling limitations, this analysis provides a conservative estimate of the extent of cross-hemisphere migration occurring during this time period.

## Results

### Extensive Viral Genetic Diversity in Australia and New Zealand

**Figure 2:**
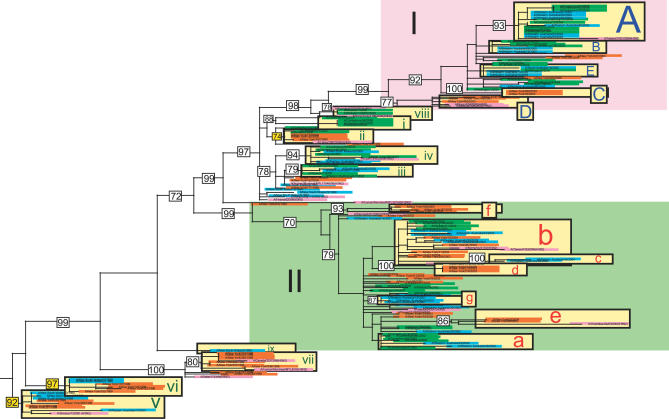
Phylogenetic Relationships of the NA Gene of A/H3N2 Influenza Viruses Sampled from New York State (*n* = 52), New Zealand (*n* = 51), Australia (*n* = 45), and Globally (*n* = 22) from 1998 to 2005, Estimated Using ML Viral isolates from New York state are highlighted in orange, isolates from New Zealand in green, isolates from Australia in blue, and global isolates in pink. Light yellow rectangles identify viral clades, with numbers in white boxes giving bootstrap values for key nodes (>70%). To clarify reassortment events, capital letters in blue refer to clades that appear in section I of the tree (denoted in pink) on the phylogeny of the concatenated six non-surface glycoproteins; lowercase letters in red refer to clades contained within section II (denoted in light green); and lowercase roman numerals in dark green refer to clades outside sections I and II. Bootstrap values highlighted in yellow identify nodes that define a cross-hemisphere migration event. The tree is rooted by isolate A/New York/327/1999 from the 1998–1999 season (i.e., the earliest sampled isolate), and all horizontal branch lengths are drawn to a scale of nucleotide substitutions per site.

**Figure 3:**
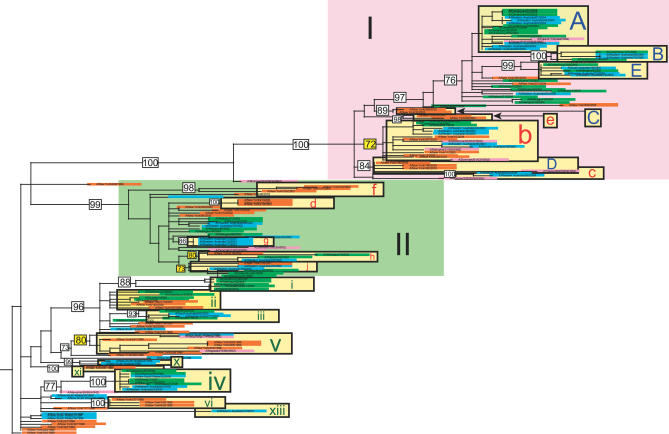
Phylogenetic Relationships of the HA Gene of A/H3N2 Influenza Viruses Sampled from New York State (*n* = 52), New Zealand (*n* = 51), Australia (*n* = 45), and Globally (*n* = 13) from 1998 to 2005, Estimated Using ML Color scheme, rooting, scale, and symbols are the same as those used in Figure 2.

**Figure 4:**
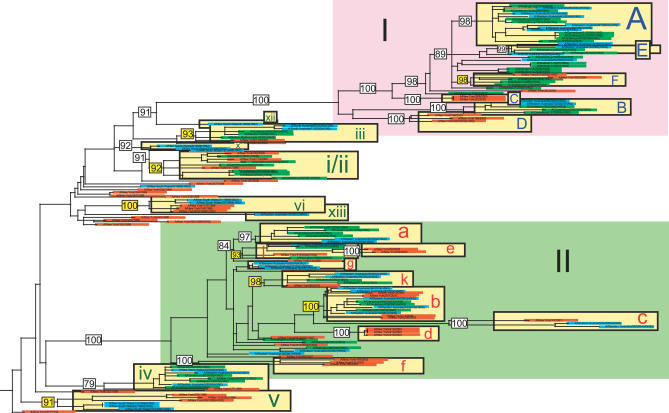
Phylogenetic Relationships of the Concatenated Six Non-Surface Glycoprotein Segments of A/H3N2 Influenza Viruses Sampled from New York State (*n* = 52), New Zealand (*n* = 51), and Australia (*n* = 45) from 1998 to 2005, Estimated Using ML Color scheme, rooting, scale, and symbols are the same as those used in Figure 2.

Populations of A/H3N2 influenza virus in Australia and New Zealand from 1999 to 2005 exhibit extensive genetic diversity across the entire genome (Figures 2–4), comparable to the diversity observed previously in New York state [7] (in all phylogenies, clades from Australia are shaded blue, those from New Zealand in green, those from New York state in orange, and global isolates in pink). In particular, multiple viral clades co-circulate during each influenza season in New Zealand and Australia, defined as clusters of isolates with high bootstrap support (>70%), or which are separated by exceptionally long branches (Table 1). As with the New York state data, these viruses were collected in the context of seasonal surveillance efforts in Australia and New Zealand and therefore likely provide a representative sample of the overall genetic composition of the viral populations.

**Table 1:**
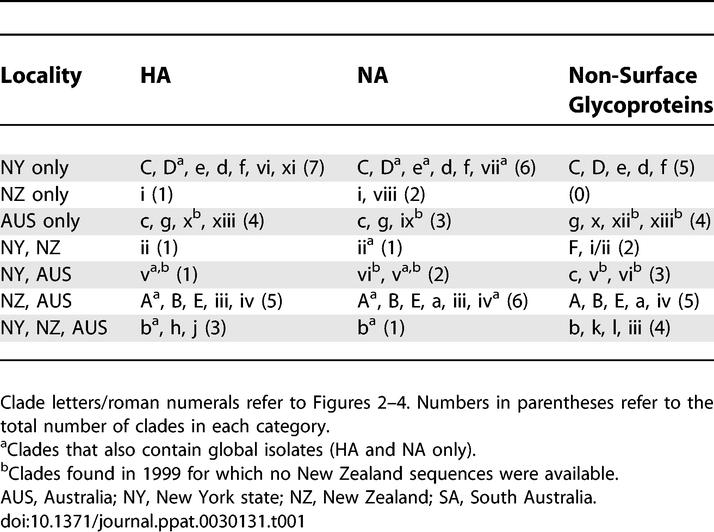
Clades in the HA, NA, and Concatenated Six Non-Surface Glycoprotein Segment Phylogenies Comprised of Viruses from New York State, New Zealand, Australia, and Globally

On the neuraminidase (NA) tree (Figure 2), viruses from New Zealand and Australia fall into at least 15 distinct clades, some of which appear in multiple seasons: three clades circulated in the 1999 season (clades v, vi, and ix; when data was only available from Australia), five in 2000 (clades i, ii, iii, iv, viii), four in 2001 (clades i, iv, v, g), one in 2002 (clade a), one in 2003 (clade b; when a new reassortant virus predominated), two in 2004 (clades A and c), and three in 2005 (clades A, B, E). Clade lettering reflects the three main sections of the phylogeny, based on topology and time: clades A–E contain viral isolates from 2003 to 2005 that fall within section I of the tree (large pink rectangle in upper portion of tree); clades a–e contain isolates from 2001–2003 that fall within section II (large light green rectangle in middle portion of tree); clades i–xiii contain isolates from 1998 to 2000 that fall outside of sections I and II. The hemagglutinin (HA) tree (Figure 3) contains at least 15 clades from New Zealand and Australia: two circulated in 1999 (clades v and x), four in 2000 (clades i, ii, iii, iv), four in 2001 (clades g, i, iv, xiii), two in 2002 (clades h and j), one in 2003 (clade b), two in 2004 (clades A and c), and three in 2005 (clades A, B, E) (Figure 3). Finally, the phylogeny of the concatenated six non-surface glycoprotein segments (PB2, PB1, PA, NP, M1, NS1) contains the largest number of clades, presumably because this larger data set (9,636 bp) provides the greatest resolution. Southern hemisphere isolates form at least 18 clades on this phylogeny: four were present in 1999 (clades v, vi, x, xii), three in 2000 (clades i/ii, iii, iv), four in 2001 (clades g, i/ii, iv, xiii), three in 2002 (clades a, k, l), one in 2003 (clade b), three in 2004 (clades A, F, c), and three in 2005 (clades A, B, E) (Figure 4).

Differences in the number of clades among segments may also be indicative of reassortment, especially involving the HA gene, as previously demonstrated in New York state [7]. Indeed, several major reassortment events are immediately evident from topological incongruities among these three phylogenies. On the HA tree, clades b, c, and e fall in section I along with isolates from 2004 and 2005, while these clades fall in section II amidst 2002 and 2003 isolates in the NA and concatenated six non-surface glycoprotein phylogenies (Figures 2–4). Clade c also falls in section I on the PB2 phylogeny, showing a similar reassortment pattern as HA. However, aside from this lone reassortment event, the phylogenies of the six non-surface glycoprotein segments are very similar, enabling us to study them as a single concatenated entity (phylogenetic trees for individual segments are provided as Figures S1–S6).

### Intermixing among Viral Populations from Australia, New Zealand, and New York State

Isolates from the 52 representative New York state genomes are clearly interspersed with southern hemisphere isolates throughout our phylogenetic trees (Figures 2–4; Table 1), indicating that these populations regularly intermix as a result of cross-hemisphere migration. However, patterns of viral intermixing are both variable and complex, as clades from all three phylogenies contain an array of different combinations of viral populations from New Zealand, Australia, and/or New York state. For example, the phylogeny of the concatenated six non-surface glycoproteins contains a relatively even mix of mono-hemisphere and bi-hemisphere clades (Table 1), with five New York state–only clades, seven southern hemisphere–only clades, six clades that contain New York state isolates and isolates from only one of the southern hemisphere countries, and three clades that contain isolates from all three countries. Thus, on an annual basis some, but not all, viral populations mix with other populations from the same and/or opposite hemisphere, and this number is likely to increase with additional sampling. Indeed, a majority (nine) of the 16 clades containing southern hemisphere isolates also contained northern hemisphere isolates, suggesting widespread viral traffic across the equator (Table 1). Migration between Australia and New Zealand is also extensive, as almost all clades containing isolates from New Zealand also contained Australian isolates, and vice versa, except for the 1999 season, for which no New Zealand isolates were available. Further, these figures are likely to be underestimates, as mono-national and mono-hemisphere clades may be at frequencies too low to be detected in the genome collections currently available. Alternatively, clades could also have originated in areas not sampled in our study, such as tropical regions, where influenza viruses typically circulate year-round [12].

Strikingly, even for those viral clades that do not exhibit cross-hemisphere migration, there is very little evidence for in situ evolution within specific localities. For example, in no case on any phylogeny are clades of A/H3N2 from New York state directly linked over multiple seasons. Rather, New Zealand and Australia viruses are always interspersed among New York state clades from different seasons, indicating that they are not evolving in geographic isolation across seasons. Most clades from New Zealand and Australia show equivalent patterns of discontinuous evolution, with very few clusters of southern hemisphere clades that are not separated by New York state viruses (although in situ evolution cannot be ruled out between a few 2004 and 2005 clades without additional sampling). Thus, even in relatively isolated areas of New Zealand and Australia, viruses do not regularly evolve in geographic isolation. Rather, evolution appears to be shaped by frequent cross-hemisphere migration and recurrent reintroduction.

### Bi-Directional Cross-Hemisphere Migration Occurs between Influenza Seasons

Importantly, our phylogenetic analysis suggests that seasonal migration occurs from the northern hemisphere to the southern hemisphere, as well as south-to-north. Although inferring the direction of viral migration is in part dependent on sample composition, definitive evidence of a migration event are clades containing a single population of northern hemisphere viruses and a single population of southern hemisphere viruses supported by a high level of bootstrap support (>70%).

Because winter influenza seasons alternate by six-month intervals between the northern and southern hemispheres, it was also possible to determine, within the confines of sampling, the timescale and hence direction of cross-hemisphere migration. The inferred directionality of 11 cases of definitive cross-hemisphere migration evident on the HA, NA, and non-surface glycoprotein phylogenies are summarized in Table 2. Of these, all but two involve the concatenated non-surface glycoproteins, which provide a more reliable phylogeny as previously described. Eight of these migration events occur in a north-to-south direction, versus three in a south-to-north direction, suggesting that viruses may migrate more frequently from New York state to the southern hemisphere than in the opposite direction, although this will need to be confirmed with larger sample sizes. For example, on the concatenated gene tree, viral isolates from the 2003–2004 season in New York state form a single well-supported phylogenetic clade (clade b, 100% bootstrap support) with viruses from 2003 from New Zealand and Australia (Figure 4). Since the 2003 New Zealand and Australia viruses predate the 2003–2004 New York state viruses (i.e., the northern hemisphere winter), we infer that the lineage that gave rise to these southern hemisphere viruses migrated northward to infect New York state between the 2003 southern hemisphere winter (May to October) and the 2003–2004 winter in New York state.

**Table 2:**
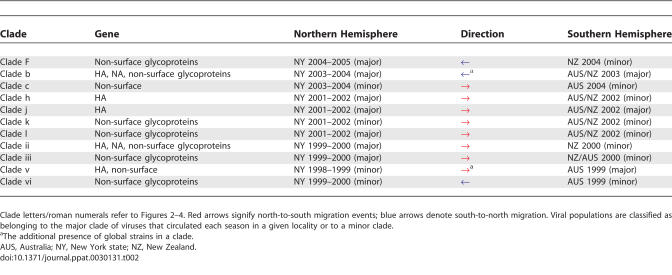
Direction of 11 Migration Events between New York State in the Northern Hemisphere and Australia and New Zealand in the Southern Hemisphere

It is also notable that cross-hemisphere migration does not follow any clear pattern. In addition to occurring in both north-to-south and south-to-north directions, migration events also appear to involve minor clades as frequently as major clades, assuming that our study sample is generally representative of the viral population structure (Table 2). Furthermore, the populations of southern hemisphere viruses that migrate northward are a mix of compositions, including some isolates only from New Zealand, some only from Australia, and populations with a mix of isolates from both countries (Tables 1 and 2). Finally, two clades contain global strains, but because the dates of these global isolates are not recorded, it is impossible to accurately determine the direction of migration events.

The 11 cases presented in Table 2 represent only the strongest examples of cross-hemisphere migration, using the strictest criteria to infer migration events from the phylogenetic data. Relaxing this stringency allows for the possibility of greater bi-directional cross-hemisphere migration, especially involving clades containing more than one viral population from the southern hemisphere. Although the direction of migration is less certain when populations of viruses from three geographical regions are present, the relative frequency of migration observed under the more stringent criteria suggests that cross-hemisphere migration likely operates in these cases as well. Finally, while it is likely that a more intensive sampling regime will increase clade diversity, and in doing so affect the inference of the direction of migration, the complexity of the patterns observed strongly argues for frequent bi-directional migration.

## Discussion

Our large-scale phylogenetic analysis of A/H3N2 influenza virus populations from opposite geographic hemispheres provides evidence for regular bi-directional cross-hemisphere viral migration between seasons, even among localities as distantly separated as New York state and Australia and as relatively geographically isolated as New Zealand's South Island. Multiple genetic variants of influenza virus co-circulate each season, even in geographically remote areas, and many of these viral clades are more closely related to isolates from the opposite hemisphere than to isolates from either the previous or following season in the same location. Thus, viral populations do not appear to “over-summer” locally, where they would evolve in situ and give rise to the next season's epidemic. Rather, cycles of viral migration and recurrent introduction have clearly played a significant role in generating the genetic diversity that characterizes influenza A virus in both hemispheres. Importantly, given the sample composition of our sequence data set, the extent of cross-hemisphere migration observed here undoubtedly represents a conservative estimate. Hence, including data from more populated areas could only reveal more instances of cross-hemisphere migration.

In addition, our finding that the virus migrates globally between epidemics and is reintroduced is clearly compatible with tropical regions, including Southeast Asia, playing a key role in the genesis of new clades and the global spread of these novel influenza virus variants. Thus, while limitations in global genome sampling necessarily means that the current study is directed toward testing hypotheses of viral migration versus latency, equivalent data from tropical regions would undoubtedly enable us to conduct a more refined analysis of global migration patterns and their determinants. Specifically, if tropical regions serve as year-long influenza reservoirs, we would expect to observe phylogenies in which tropical isolates display the greatest genetic diversity and are positioned basal to viruses sampled from temperate regions. Consequently, complete genome sampling from tropical regions where influenza viruses circulate year-round, including a record of the precise date of collection, is of key importance for understanding the global epidemiology of the influenza virus.

Notably, the viral migration we observe does not appear to follow any clear pattern, but rather occurs in all directions, involves all genes, and involves clades of all sizes and geographic compositions. This argues against a role of immune selection in determining which viral clades are able to migrate among localities, although it does not preclude a role for natural selection as the sieve that determines which clades are able to survive in specific host populations. Similarly, the observation that migration patterns vary to some extent among the HA, NA, and concatenated non-surface glycoproteins must reflect the effect of widespread genomic reassortment [7,20]. Frequent reassortment complicates the analysis of migration patterns, as individual viruses can carry genomic segments with differing phylogenetic, and hence geographical, histories. Consequently, the analysis of migration patterns based on single gene segments may paint a misleading picture.

Although the transmission of the influenza virus through population movements has been studied extensively, particularly for the spread of pandemic isolates across the globe by air travel [21,22], neither the routes nor the mechanisms of the virus's geographical spread have been fully resolved. Several recent studies have used empirical data to investigate the role of population movements on the spatial diffusion of seasonal epidemics, including an intricate analysis of the regional spread of influenza epidemics across the United States, which was strongly correlated with adult workflow movements [23]. A previous epidemiological study comparing the synchronicity with respect to timing of influenza epidemics between the United States, France, and Australia suggested that the inter-hemispheric circulation of epidemics follows an irregular pathway, with recurrent changes in the leading hemisphere [24], in accordance with the phylogenetic analysis presented here. More fine-scaled analyses of discrete viral populations have shown that frequent introduction of “foreign” viruses significantly impacts the viral population structure and geographic spread at local levels. For example, the rapid timescale of global mixing of influenza drowns out any impact of local heterogeneities on the spread of the epidemics through France [25]. Similarly, the seasonal importation of multiple global isolates appears to be a greater contributor to the genetic diversity of the influenza virus population in New York state from 1997 to 2005 than local in situ evolution [7]. While our findings confirm that human population movements play a role in introducing new viral variants at the start of an epidemic, some aspect of climate is clearly of importance in triggering epidemics. Additional research is required to define how human susceptibility to infection and viral transmissibility fluctuate under varying climate conditions and why influenza virus is absent in summer in temperate climates but exists year-round in tropical zones.

Although the underlying cause of the seasonality of the influenza virus remains uncertain, even in reservoir avian species [26], our findings illustrate the critical importance of expanding surveillance to elucidate the geographical movements and evolution of this virus throughout its entire annual cycle. The traditional focus on epidemic influenza may detract from the equally important epidemiological question of why influenza A virus does not circulate in humans for so many months of the year in temperate areas, especially given its apparent ability to infect humans in tropical areas year-round. Attempts to predict, model, or contain the spread of the influenza virus require a unified understanding of how the virus's spatial-temporal dynamics, antigenic evolution, and seasonal emergence interrelate [27]. Although this study is limited to only the three countries for which we have extensive data, our analysis exemplifies the capacity of phylogenetic analysis to elucidate challenging epidemiological questions by providing a level of finer resolution.

## Materials and Methods

### Influenza viruses used in this study.

All influenza A (H3N2) virus complete genome sequence data were collected from the National Institute of Allergy and Infectious Disease's Influenza Genome Sequencing Project (http://www.niaid.nih.gov/dmid/genomes/mscs/influenza.htm) for the period 1998–2005 [28]. Influenza A/H3N2 viruses were sampled by a network of participating general practitioners. Viruses from all 11 regions in New York state were collected by the Virus Reference and Surveillance Laboratory at the Wadsworth Center, New York State Department of Health. Influenza viruses from both the North and South Islands of New Zealand were collected by Canterbury Health Laboratories in Christchurch, New Zealand. In Australia, viruses from Western Australia were collected by PathWest Laboratory Medicine, Western Australia; viruses from New South Wales were collected by the Prince of Wales Hospital, New South Wales; viruses from South Australia were collected by the Institute of Medical and Veterinary Sciences, South Australia; and viruses from Queensland were collected by the Queensland Health Science Services, Queensland. All sequence data were downloaded from the National Center for Biotechnology Information (NCBI) Influenza Virus Resource (http://www.ncbi.nlm.nih.gov/genomes/FLU/FLU.html). For Australia, 88 genome sequences from the 1999–2005 seasons were compiled, while for New Zealand, 399 genome sequences A/H3N2 sequences from the 2000–2005 seasons were collected. For New York state, United States, 52 phylogenetically representative genome sequences from the 1998–1999 to 2004–2005 seasons were carefully selected from a larger data set of 413 sequences from 1997–2005 analyzed previously [7] (excluding 2000–2001, for which few H3N2 sequences were available in an H1N1-dominant season). GenBank accession numbers for all sequences used in this study are listed in Table S1.

### Phylogenetic analysis.

Sequence alignments were manually constructed for the major coding regions of each of the eight genomic segments. In addition to alignments for the HA (1,698 bp) and NA (1,407 bp), an alignment of the concatenated six non-surface glycoproteins segments (PB2, PB1, PA, NP, M1, NS1) was also compiled (9,636 bp), as these are expected to evolve differently from the HA and NA surface glycoproteins. Because the minor M2 and NS2 proteins are involved in overlapping reading frames, they were excluded from the analysis.

Initial phylogenetic trees were inferred for sequences of the HA, NA, and concatenated non-surface glycoproteins from New York state, New Zealand, and Australia under the HKY85 (Hasegawa-Kishino-Yano) model of nucleotide substitution using the Neighbor-Joining (NJ) method available in PAUP* [29]. Due to the very large size of all data sets, and the provisional nature of the analysis, the nearest-neighbor-interchange branch-swapping method was employed in this case. To assess the robustness of individual nodes on these phylogenetic trees, we performed a bootstrap resampling analysis (1,000 replications) using the NJ method. From these three starting phylogenetic trees, “major” clades (which contained the majority of isolates from a season) and “minor” clades of genetically related viruses were identified by exceptionally long branch lengths and/or high bootstrap values (>70%). A subset of sequences for the concatenated non-surface glycoproteins was constructed with 51 sequences from New Zealand, 45 sequences from Australia, and 52 from New York state (see above) for a total data set of 148 sequences. For the HA gene, these 148 isolates were placed in a more global context with the addition of 13 genetically unique HA sequences sampled from this time period available on GenBank, to produce a total of 161 HA sequences. Likewise, 22 global NA sequences were combined with the original 148 from New York state, New Zealand, and Australia for a total of 170 NA sequences. Maximum likelihood (ML) phylogenetic trees were then inferred using the PAUP* package [29] for these three new data sets: 161 HA sequences, 170 NA sequences, 148 concatenated sequences. ML trees were also inferred for each of the six non-surface glycoprotein segments to ensure that all exhibit similar tree topologies (Figures S1–S6). In each case, the best-fit model of nucleotide substitution was identified by MODELTEST [30] as the general reversible GTR+I+Γ_4_ model, with the frequency of each substitution type, proportion of invariant sites (I), and the gamma distribution (Γ) of among-site rate variation with four rate categories (Γ_4_) estimated from the empirical data. In all cases tree bisection-reconnection branch-swapping was utilized to determine the optimal tree. Finally, a bootstrap resampling process (1,000 replications) using the NJ method was used to assess the robustness of individual nodes on the phylogeny, incorporating the ML substitution model.

The analysis of the frequency and directionality of migration was undertaken through a visual inspection of the topological position of individual clades on each tree and in consideration of their time of sampling. Although more quantitative methods for determining migration patterns from gene sequence data have been established, particularly those based on parsimony reconstructions of changes in character state (i.e., geographical locality) [31], these were considered inappropriate for the current study because they ignore the temporal structure of the influenza virus genome sequence data. Specifically, we reasonably assume that older sampled clades give rise to younger sampled clades if they fall basal to them on phylogenetic trees.

## Supporting Information

Figure S1 Phylogenetic Relationships of the PB2 Gene Segment of A/H3N2 Influenza Viruses Sampled from New York State (*n* = 52), New Zealand (*n* = 51), and Australia (*n* = 45) from 1998–2005, Estimated Using ML Method Viral isolates from New York state are highlighted in orange, isolates from New Zealand in green, isolates from Australia in blue, and global isolates in pink. Light yellow rectangles identify viral clades, with numbers in white boxes giving bootstrap values for key nodes (>70%). Capital letters in blue refer to clades that appear in section I of the tree (denoted in pink) on the phylogeny of the concatenated six non-surface glycoprotein segments; lowercase letters in red refer to clades contained within section II (denoted in light green); lowercase roman numerals in dark green refer to clades outside sections I and II. Bootstrap values highlighted in yellow identify nodes that define a cross-hemisphere migration event. The tree is rooted by isolate A/New York/327/1999 from the 1998–1999 season (i.e., the earliest sampled isolate), and all horizontal branch lengths are drawn to a scale of nucleotide substitutions per site.
(975 KB EPS)

Figure S2 Phylogenetic Relationships of the PB1 Gene Segment of A/H3N2 Influenza Viruses Sampled from New York State (*n* = 52), New Zealand (*n* = 51), and Australia (*n* = 45) from 1998–2005, Estimated Using ML Method Color scheme, rooting, scale, and symbols are the same as those used in Figure S1.
(960 KB EPS)

Figure S3 Phylogenetic Relationships of the PA Gene Segment of A/H3N2 Influenza Viruses Sampled from New York State (*n* = 52), New Zealand (*n* = 51), and Australia (*n* = 45) from 1998–2005, Estimated Using ML Method Color scheme, rooting, scale, and symbols are the same as those used in Figure S1.
(970 KB EPS)

Figure S4 Phylogenetic Relationships of the NP Gene of A/H3N2 Influenza Viruses Sampled from New York State (*n* = 52), New Zealand (*n* = 51), and Australia (*n* = 45) from 1998–2005, Estimated Using ML Method Color scheme, rooting, scale, and symbols are the same as those used in Figure S1.
(951 KB EPS)

Figure S5 Phylogenetic Relationships of the M1 Gene of A/H3N2 Influenza Viruses Sampled from New York State (*n* = 52), New Zealand (*n* = 51), and Australia (*n* = 45) from 1998–2005, Estimated Using ML Method Color scheme, rooting, scale, and symbols are the same as those used in Figure S1.
(3.9 MB EPS)

Figure S6 Phylogenetic Relationships of the NS1 Gene of A/H3N2 Influenza Viruses Sampled from New York State (*n* = 52), New Zealand (*n* = 51), and Australia (*n* = 45) from 1998–2005, Estimated Using ML Method Color scheme, rooting, scale, and symbols are the same as those used in Figure S1.
(830 KB EPS)

Table S1 Complete Genome Sequences of A/H3N2 Influenza Viruses Sampled from Australia, New Zealand, and New York State Used in This Study (535 KB DOC)

## Abbreviations

HA: hemagglutinin
ML: maximum likelihood
NA: neuraminidase
NJ: Neighbor-Joining
