# Supplementary material for: Phylogenetic Analysis Reveals the Global Migration of Seasonal Influenza A Viruses
Source: PLoS Pathog. 2007 Sep 14;3(9):e131. doi: 10.1371/journal.ppat.0030131 (PMC2323296; doi:10.1371/journal.ppat.0030131)
Supplement: Table S1 — (535 KB DOC) [file ppat.0030131.st001.doc]

**Table S1.** Complete genome sequences of A/H3N2 influenza viruses from Australia, New Zealand, and New York State used in this study.

All viral genome sequences were downloaded from the Influenza Virus Resource available through GenBank (<http://www.ncbi.nlm.nih.gov/genomes/FLU/FLU.html>).

**A. 399 whole genome sequences of H3N2 influenza A virus from New Zealand, 2000 – 2005 (GenBank accession numbers correspond to PB2 gene).**

|  | Accession | Isolate |  | Accession | Isolate |
| --- | --- | --- | --- | --- | --- |
| 1 | CY008851 | A/Canterbury/101/2000 | 201 | CY012095 | A/Waikato/102/2003 |
| 2 | CY008843 | A/Canterbury/103/2000 | 202 | CY013444 | A/Waikato/108/2003 |
| 3 | CY008483 | A/Canterbury/17/2000 | 203 | CY012375 | A/Waikato/115/2003 |
| 4 | CY009123 | A/Canterbury/2/2000 | 204 | CY011775 | A/Waikato/120/2003 |
| 5 | CY009107 | A/Canterbury/3/2000 | 205 | CY012391 | A/Waikato/122/2003 |
| 6 | CY008491 | A/Canterbury/38/2000 | 206 | CY012399 | A/Waikato/129/2003 |
| 7 | CY008146 | A/Canterbury/39/2000 | 207 | CY012407 | A/Waikato/133/2003 |
| 8 | CY008138 | A/Canterbury/42/2000 | 208 | CY012415 | A/Waikato/139/2003 |
| 9 | CY009139 | A/Canterbury/55/2000 | 209 | CY012423 | A/Waikato/147/2003 |
| 10 | CY008755 | A/Canterbury/56/2000 | 210 | CY012431 | A/Waikato/148/2003 |
| 11 | CY009155 | A/Canterbury/58/2000 | 211 | CY013436 | A/Waikato/15/2003 |
| 12 | CY009171 | A/Canterbury/61/2000 | 212 | CY012103 | A/Waikato/150/2003 |
| 13 | CY011071 | A/Canterbury/62/2000 | 213 | CY012079 | A/Waikato/154/2003 |
| 14 | CY009147 | A/Canterbury/64/2000 | 214 | CY012351 | A/Waikato/155/2003 |
| 15 | CY008771 | A/Canterbury/66/2000 | 215 | CY012359 | A/Waikato/156/2003 |
| 16 | CY009163 | A/Canterbury/67/2000 | 216 | CY013119 | A/Waikato/21/2003 |
| 17 | CY008763 | A/Canterbury/68/2000 | 217 | CY012367 | A/Waikato/29/2003 |
| 18 | CY008779 | A/Canterbury/71/2000 | 218 | CY011735 | A/Waikato/3/2003 |
| 19 | CY008499 | A/Canterbury/73/2000 | 219 | CY013127 | A/Waikato/46/2003 |
| 20 | CY008787 | A/Canterbury/80/2000 | 220 | CY013135 | A/Waikato/53/2003 |
| 21 | CY008795 | A/Canterbury/81/2000 | 221 | CY013143 | A/Waikato/54/2003 |
| 22 | CY008803 | A/Canterbury/84/2000 | 222 | CY011759 | A/Waikato/61/2003 |
| 23 | CY008811 | A/Canterbury/85/2000 | 223 | CY011767 | A/Waikato/75/2003 |
| 24 | CY009083 | A/Canterbury/87/2000 | 224 | CY012087 | A/Waikato/91/2003 |
| 25 | CY008507 | A/Canterbury/88/2000 | 225 | CY013151 | A/Waikato/94/2003 |
| 26 | CY008827 | A/Canterbury/89/2000 | 226 | CY011727 | A/Wellington/10/2003 |
| 27 | CY008867 | A/Canterbury/90/2000 | 227 | CY011743 | A/Wellington/25/2003 |
| 28 | CY009099 | A/Canterbury/92/2000 | 228 | CY011751 | A/Wellington/28/2003 |
| 29 | CY008835 | A/Canterbury/93/2000 | 229 | CY011703 | A/Wellington/3/2003 |
| 30 | CY009395 | A/Canterbury/94/2000 | 230 | CY012071 | A/Wellington/34/2003 |
| 31 | CY009091 | A/Canterbury/96/2000 | 231 | CY011711 | A/Wellington/4/2003 |
| 32 | CY012439 | A/Canterbury/97/2000 | 232 | CY012663 | A/Wellington/47/2003 |
| 33 | CY008859 | A/Canterbury/98/2000 | 233 | CY012671 | A/Wellington/49/2003 |
| 34 | CY008515 | A/Canterbury/99/2000 | 234 | CY012383 | A/Wellington/53/2003 |
| 35 | CY012623 | A/Dunedin/1/2000 | 235 | CY012655 | A/Wellington/9/2003 |
| 36 | CY013382 | A/Dunedin/3/2000 | 236 | CY002961 | A/Ashburton/280/2004 |
| 37 | CY008819 | A/Hutt/82/2000 | 237 | CY007298 | A/Bay of Plenty/279/2004 |
| 38 | CY009131 | A/Nelson Marlborough/1/2000 | 238 | CY007306 | A/Bay of Plenty/332/2004 |
| 39 | CY011599 | A/Waikato/1/2000 | 239 | CY007322 | A/Bay of Plenty/383/2004 |
| 40 | CY013862 | A/Waikato/15/2000 | 240 | CY007426 | A/Canterbury/100/2004 |
| 41 | CY013396 | A/Waikato/16/2000 | 241 | CY007434 | A/Canterbury/101/2004 |
| 42 | CY013404 | A/Waikato/17/2000 | 242 | CY007442 | A/Canterbury/102/2004 |
| 43 | CY013071 | A/Waikato/20/2000 | 243 | CY007450 | A/Canterbury/103/2004 |
| 44 | CY012631 | A/Waikato/4/2000 | 244 | CY007458 | A/Canterbury/104/2004 |
| 45 | CY011967 | A/Waikato/5/2000 | 245 | CY007466 | A/Canterbury/105/2004 |
| 46 | CY013055 | A/Waikato/6/2000 | 246 | CY007482 | A/Canterbury/107/2004 |
| 47 | CY012639 | A/Waikato/7/2000 | 247 | CY007994 | A/Canterbury/108/2004 |
| 48 | CY013063 | A/Waikato/9/2000 | 248 | CY008002 | A/Canterbury/109/2004 |
| 49 | CY013910 | A/Wellington/30/2000 | 249 | CY007346 | A/Canterbury/11/2004 |
| 50 | CY011027 | A/Wellington/9/2000 | 250 | CY007354 | A/Canterbury/12/2004 |
| 51 | CY009955 | A/Canterbury/06/2001 | 251 | CY007362 | A/Canterbury/16/2004 |
| 52 | CY009563 | A/Canterbury/07/2001 | 252 | CY007370 | A/Canterbury/17/2004 |
| 53 | CY009403 | A/Canterbury/10/2001 | 253 | CY007378 | A/Canterbury/18/2004 |
| 54 | CY009595 | A/Canterbury/140/2001 | 254 | CY007386 | A/Canterbury/19/2004 |
| 55 | CY009859 | A/Canterbury/146/2001 | 255 | CY007394 | A/Canterbury/20/2004 |
| 56 | CY009443 | A/Canterbury/149/2001 | 256 | CY007490 | A/Canterbury/201/2004 |
| 57 | CY010555 | A/Canterbury/36/2001 | 257 | CY007498 | A/Canterbury/202/2004 |
| 58 | CY009419 | A/Canterbury/37/2001 | 258 | CY007506 | A/Canterbury/205/2004 |
| 59 | CY009579 | A/Canterbury/43/2001 | 259 | CY007514 | A/Canterbury/206/2004 |
| 60 | CY009427 | A/Canterbury/44/2001 | 260 | CY007522 | A/Canterbury/207/2004 |
| 61 | CY009587 | A/Canterbury/50/2001 | 261 | CY007530 | A/Canterbury/208/2004 |
| 62 | CY011975 | A/Waikato/1/2001 | 262 | CY007538 | A/Canterbury/209/2004 |
| 63 | CY012327 | A/Waikato/105/2001 | 263 | CY007402 | A/Canterbury/21/2004 |
| 64 | CY013079 | A/Waikato/5/2001 | 264 | CY007546 | A/Canterbury/210/2004 |
| 65 | CY011623 | A/Wellington/22/2001 | 265 | CY007410 | A/Canterbury/23/2004 |
| 66 | CY012319 | A/Wellington/34/2001 | 266 | CY007418 | A/Canterbury/24/2004 |
| 67 | CY009571 | A/West Coast/28/2001 | 267 | CY007554 | A/Canterbury/303/2004 |
| 68 | CY009411 | A/West Coast/32/2001 | 268 | CY007562 | A/Canterbury/304/2004 |
| 69 | CY009435 | A/West Coast/55/2001 | 269 | CY008227 | A/Canterbury/305/2004 |
| 70 | CY007594 | A/Canterbury/01/2002 | 270 | CY008235 | A/Canterbury/308/2004 |
| 71 | CY007602 | A/Canterbury/02/2002 | 271 | CY008243 | A/Canterbury/309/2004 |
| 72 | CY008010 | A/Canterbury/04/2002 | 272 | CY008251 | A/Canterbury/310/2004 |
| 73 | CY007610 | A/Canterbury/05/2002 | 273 | CY007570 | A/Canterbury/311/2004 |
| 74 | CY008267 | A/Canterbury/06/2002 | 274 | CY008259 | A/Canterbury/312/2004 |
| 75 | CY008275 | A/Canterbury/09/2002 | 275 | CY007578 | A/Canterbury/313/2004 |
| 76 | CY008283 | A/Canterbury/10/2002 | 276 | CY007586 | A/Canterbury/315/2004 |
| 77 | CY007794 | A/Canterbury/102/2002 | 277 | CY002976 | A/Christchurch/10/2004 |
| 78 | CY008018 | A/Canterbury/13/2002 | 278 | CY002913 | A/Christchurch/13/2004 |
| 79 | CY007850 | A/Canterbury/14/2002 | 279 | CY002929 | A/Christchurch/14/2004 |
| 80 | CY008291 | A/Canterbury/15/2002 | 280 | CY002921 | A/Christchurch/15/2004 |
| 81 | CY007658 | A/Canterbury/16/2002 | 281 | CY002953 | A/Christchurch/184/2004 |
| 82 | CY007666 | A/Canterbury/18/2002 | 282 | CY002969 | A/Christchurch/297/2004 |
| 83 | CY007858 | A/Canterbury/19/2002 | 283 | CY002983 | A/Christchurch/339/2004 |
| 84 | CY007866 | A/Canterbury/20/2002 | 284 | CY002937 | A/Christchurch/89/2004 |
| 85 | CY008026 | A/Canterbury/21/2002 | 285 | CY002945 | A/Christchurch/90/2004 |
| 86 | CY007674 | A/Canterbury/22/2002 | 286 | CY008219 | A/Tairawhiti/223/2004 |
| 87 | CY007874 | A/Canterbury/27/2002 | 287 | CY007314 | A/Tairawhiti/369/2004 |
| 88 | CY007882 | A/Canterbury/29/2002 | 288 | CY013468 | A/Waikato/12/2004 |
| 89 | CY007890 | A/Canterbury/31/2002 | 289 | CY012711 | A/Waikato/21/2004 |
| 90 | CY007898 | A/Canterbury/33/2002 | 290 | CY013508 | A/Waikato/22/2004 |
| 91 | CY007906 | A/Canterbury/34/2002 | 291 | CY013516 | A/Waikato/26/2004 |
| 92 | CY007682 | A/Canterbury/35/2002 | 292 | CY013926 | A/Waikato/3/2004 |
| 93 | CY007690 | A/Canterbury/41/2002 | 293 | CY013532 | A/Waikato/35/2004 |
| 94 | CY007922 | A/Canterbury/44/2002 | 294 | CY012727 | A/Waikato/40/2004 |
| 95 | CY008034 | A/Canterbury/46/2002 | 295 | CY013540 | A/Waikato/43/2004 |
| 96 | CY007698 | A/Canterbury/47/2002 | 296 | CY013548 | A/Waikato/45/2004 |
| 97 | CY007706 | A/Canterbury/48/2002 | 297 | CY013942 | A/Waikato/51/2004 |
| 98 | CY007714 | A/Canterbury/49/2002 | 298 | CY013950 | A/Waikato/56/2004 |
| 99 | CY007722 | A/Canterbury/50/2002 | 299 | CY013958 | A/Waikato/64/2004 |
| 100 | CY007730 | A/Canterbury/53/2002 | 300 | CY013966 | A/Waikato/68/2004 |
| 101 | CY007738 | A/Canterbury/56/2002 | 301 | CY013974 | A/Waikato/69/2004 |
| 102 | CY008042 | A/Canterbury/57/2002 | 302 | CY013982 | A/Waikato/71/2004 |
| 103 | CY007930 | A/Canterbury/58/2002 | 303 | CY013998 | A/Waikato/72/2004 |
| 104 | CY007746 | A/Canterbury/59/2002 | 304 | CY014006 | A/Waikato/73/2004 |
| 105 | CY008299 | A/Canterbury/60/2002 | 305 | CY012111 | A/Wellington/1/2004 |
| 106 | CY008307 | A/Canterbury/61/2002 | 306 | CY012679 | A/Wellington/14/2004 |
| 107 | CY008315 | A/Canterbury/62/2002 | 307 | CY013476 | A/Wellington/18/2004 |
| 108 | CY008323 | A/Canterbury/64/2002 | 308 | CY012695 | A/Wellington/22/2004 |
| 109 | CY007938 | A/Canterbury/66/2002 | 309 | CY013460 | A/Wellington/23/2004 |
| 110 | CY007754 | A/Canterbury/68/2002 | 310 | CY013484 | A/Wellington/27/2004 |
| 111 | CY007762 | A/Canterbury/69/2002 | 311 | CY012703 | A/Wellington/31/2004 |
| 112 | CY008331 | A/Canterbury/70/2002 | 312 | CY013159 | A/Wellington/35/2004 |
| 113 | CY007946 | A/Canterbury/72/2002 | 313 | CY013500 | A/Wellington/38/2004 |
| 114 | CY007770 | A/Canterbury/75/2002 | 314 | CY013918 | A/Wellington/4/2004 |
| 115 | CY008339 | A/Canterbury/76/2002 | 315 | CY013492 | A/Wellington/44/2004 |
| 116 | CY007954 | A/Canterbury/79/2002 | 316 | CY013167 | A/Wellington/45/2004 |
| 117 | CY007778 | A/Canterbury/80/2002 | 317 | CY012719 | A/Wellington/52/2004 |
| 118 | CY007786 | A/Canterbury/81/2002 | 318 | CY013524 | A/Wellington/58/2004 |
| 119 | CY011407 | A/Dunedin/10/2002 | 319 | CY013934 | A/Wellington/59/2004 |
| 120 | CY013428 | A/Dunedin/12/2002 | 320 | CY012119 | A/Wellington/6/2004 |
| 121 | CY011663 | A/Dunedin/14/2002 | 321 | CY013556 | A/Wellington/62/2004 |
| 122 | CY013111 | A/Dunedin/18/2002 | 322 | CY013990 | A/Wellington/64/2004 |
| 123 | CY012647 | A/Dunedin/3/2002 | 323 | CY012687 | A/Wellington/8/2004 |
| 124 | CY012015 | A/Dunedin/8/2002 | 324 | CY009931 | A/Whanganui/127/2004 |
| 125 | CY012007 | A/Dunedin/9/2002 | 325 | CY007282 | A/Whanganui/128/2004 |
| 126 | CY007914 | A/South Canterbury/37/2002 | 326 | CY007290 | A/Whanganui/129/2004 |
| 127 | CY011983 | A/Waikato/2/2002 | 327 | CY007330 | A/Whanganui/386/2004 |
| 128 | CY011655 | A/Waikato/21/2002 | 328 | CY007338 | A/Whanganui/417/2004 |
| 129 | CY015563 | A/Waikato/25/2002 | 329 | CY008211 | A/Whanganui/69/2004 |
| 130 | CY013095 | A/Waikato/29/2002 | 330 | CY016219 | A/Waikato/1/2004 |
| 131 | CY012031 | A/Waikato/31/2002 | 331 | CY015571 | A/Waikato/16/2004 |
| 132 | CY013420 | A/Waikato/36/2002 | 332 | CY015579 | A/Wellington/34/2004 |
| 133 | CY013103 | A/Waikato/5/2002 | 333 | CY007802 | A/Canterbury/01/2005 |
| 134 | CY011631 | A/Waikato/51/2002 | 334 | CY007810 | A/Canterbury/02/2005 |
| 135 | CY012063 | A/Waikato/52/2002 | 335 | CY007818 | A/Canterbury/03/2005 |
| 136 | CY012055 | A/Waikato/58/2002 | 336 | CY008363 | A/Canterbury/104/2005 |
| 137 | CY011671 | A/Wellington/38/2002 | 337 | CY008563 | A/Canterbury/105/2005 |
| 138 | CY011639 | A/Wellington/6/2002 | 338 | CY009051 | A/Canterbury/124/2005 |
| 139 | CY011647 | A/Wellington/66/2002 | 339 | CY009939 | A/Canterbury/125/2005 |
| 140 | CY012335 | A/Wellington/7/2002 | 340 | CY008074 | A/Canterbury/127/2005 |
| 141 | CY012023 | A/Wellington/71/2002 | 341 | CY008082 | A/Canterbury/129/2005 |
| 142 | CY012047 | A/Wellington/79/2002 | 342 | CY007962 | A/Canterbury/16/2005 |
| 143 | CY012039 | A/Wellington/80/2002 | 343 | CY008090 | A/Canterbury/166/2005 |
| 144 | CY011695 | A/Wellington/86/2002 | 344 | CY008371 | A/Canterbury/186/2005 |
| 145 | CY012336 | A/Wellington/63/2002 | 345 | CY008347 | A/Canterbury/20/2005 |
| 146 | CY011672 | A/Wellington/83/2002 | 346 | CY008379 | A/Canterbury/204/2005 |
| 147 | CY013087 | A/Wellington/9/2002 | 347 | CY008571 | A/Canterbury/205/2005 |
| 148 | CY011999 | A/Wellington/70/2002 | 348 | CY008098 | A/Canterbury/206/2005 |
| 149 | CY006930 | A/Canterbury/382/2003 | 349 | CY008106 | A/Canterbury/212/2005 |
| 150 | CY006938 | A/Canterbury/384/2003 | 350 | CY008387 | A/Canterbury/220/2005 |
| 151 | CY006946 | A/Canterbury/386/2003 | 351 | CY008579 | A/Canterbury/230/2005 |
| 152 | CY006954 | A/Canterbury/387/2003 | 352 | CY013247 | A/Canterbury/232/2005 |
| 153 | CY006962 | A/Canterbury/390/2003 | 353 | CY008587 | A/Canterbury/233/2005 |
| 154 | CY006970 | A/Canterbury/391/2003 | 354 | CY008395 | A/Canterbury/234/2005 |
| 155 | CY006978 | A/Canterbury/392/2003 | 355 | CY008403 | A/Canterbury/235/2005 |
| 156 | CY006986 | A/Canterbury/393/2003 | 356 | CY008411 | A/Canterbury/236/2005 |
| 157 | CY006994 | A/Canterbury/394/2003 | 357 | CY008419 | A/Canterbury/237/2005 |
| 158 | CY007002 | A/Canterbury/395/2003 | 358 | CY008427 | A/Canterbury/238/2005 |
| 159 | CY007010 | A/Canterbury/397/2003 | 359 | CY008050 | A/Canterbury/24/2005 |
| 160 | CY007018 | A/Canterbury/398/2003 | 360 | CY008435 | A/Canterbury/242/2005 |
| 161 | CY007026 | A/Canterbury/399/2003 | 361 | CY008595 | A/Canterbury/248/2005 |
| 162 | CY009027 | A/Canterbury/400/2003 | 362 | CY010091 | A/Canterbury/250/2005 |
| 163 | CY007034 | A/Canterbury/401/2003 | 363 | CY008603 | A/Canterbury/251/2005 |
| 164 | CY007042 | A/Canterbury/403/2003 | 364 | CY008611 | A/Canterbury/253/2005 |
| 165 | CY007050 | A/Canterbury/404/2003 | 365 | CY008619 | A/Canterbury/255/2005 |
| 166 | CY007058 | A/Canterbury/405/2003 | 366 | CY008627 | A/Canterbury/256/2005 |
| 167 | CY007066 | A/Canterbury/406/2003 | 367 | CY008635 | A/Canterbury/257/2005 |
| 168 | CY007074 | A/Canterbury/408/2003 | 368 | CY008643 | A/Canterbury/258/2005 |
| 169 | CY007826 | A/Canterbury/409/2003 | 369 | CY008114 | A/Canterbury/259/2005 |
| 170 | CY007082 | A/Canterbury/410/2003 | 370 | CY009035 | A/Canterbury/26/2005 |
| 171 | CY007090 | A/Canterbury/411/2003 | 371 | CY008443 | A/Canterbury/260/2005 |
| 172 | CY007098 | A/Canterbury/412/2003 | 372 | CY008651 | A/Canterbury/266/2005 |
| 173 | CY007106 | A/Canterbury/416/2003 | 373 | CY008451 | A/Canterbury/269/2005 |
| 174 | CY007114 | A/Canterbury/417/2003 | 374 | CY008659 | A/Canterbury/270/2005 |
| 175 | CY007122 | A/Canterbury/418/2003 | 375 | CY008058 | A/Canterbury/29/2005 |
| 176 | CY007130 | A/Canterbury/420/2003 | 376 | CY007970 | A/Canterbury/33/2005 |
| 177 | CY007138 | A/Canterbury/423/2003 | 377 | CY009043 | A/Canterbury/34/2005 |
| 178 | CY007146 | A/Canterbury/424/2003 | 378 | CY008355 | A/Canterbury/64/2005 |
| 179 | CY008547 | A/Canterbury/425/2003 | 379 | CY008066 | A/Canterbury/67/2005 |
| 180 | CY008555 | A/Canterbury/426/2003 | 380 | CY014046 | A/Otago/1/2005 |
| 181 | CY007154 | A/Canterbury/427/2003 | 381 | CY014094 | A/Otago/2/2005 |
| 182 | CY007162 | A/Canterbury/428/2003 | 382 | CY014134 | A/Otago/3/2005 |
| 183 | CY007834 | A/Canterbury/429/2003 | 383 | CY014126 | A/Otago/4/2005 |
| 184 | CY007170 | A/Canterbury/430/2003 | 384 | CY014062 | A/Southland/5/2005 |
| 185 | CY007178 | A/Canterbury/431/2003 | 385 | CY014022 | A/Waikato/1/2005 |
| 186 | CY008203 | A/Canterbury/432/2003 | 386 | CY014142 | A/Waikato/12/2005 |
| 187 | CY007194 | A/Canterbury/434/2003 | 387 | CY015595 | A/Waikato/2/2005 |
| 188 | CY007202 | A/Canterbury/435/2003 | 388 | CY015603 | A/Waikato/3/2005 |
| 189 | CY007210 | A/Canterbury/436/2003 | 389 | CY014078 | A/Waikato/7/2005 |
| 190 | CY007218 | A/Canterbury/437/2003 | 390 | CY014118 | A/Waikato/9/2005 |
| 191 | CY007226 | A/Canterbury/438/2003 | 391 | CY014030 | A/Wellington/1/2005 |
| 192 | CY007234 | A/Canterbury/439/2003 | 392 | CY014038 | A/Wellington/2/2005 |
| 193 | CY007242 | A/Canterbury/440/2003 | 393 | CY014054 | A/Wellington/3/2005 |
| 194 | CY007250 | A/Canterbury/441/2003 | 394 | CY014102 | A/Wellington/8/2005 |
| 195 | CY007258 | A/Canterbury/442/2003 | 395 | CY014110 | A/Wellington/9/2005 |
| 196 | CY007266 | A/Canterbury/443/2003 | 396 | CY015635 | A/Waikato/15/2005 |
| 197 | CY007274 | A/Canterbury/444/2003 | 397 | CY015611 | A/Wellington/4/2005 |
| 198 | CY013452 | A/Dunedin/38/2003 | 398 | CY015619 | A/Wellington/5/2005 |
| 199 | CY007186 | A/South Canterbury/433/2003 | 399 | CY015627 | A/Wellington/6/2005 |
| 200 | CY011719 | A/Waikato/1/2003 |  |  |  |

**B. 88 whole genome sequences of H3N2 influenza A virus from Australia, 1999 – 2005 (GenBank accession numbers correspond to PB2 gene).**

|  | Accession | Isolate |  | Accession | Isolate |
| --- | --- | --- | --- | --- | --- |
| 1 | CY016068 | A/New South Wales/13/1999 | 45 | CY015740 | A/Western Australia/30/2002 |
| 2 | CY016076 | A/New South Wales/20/1999 | 46 | CY015748 | A/Western Australia/31/2002 |
| 3 | CY016507 | A/New South Wales/2/1999 | 47 | CY015756 | A/Western Australia/35/2002 |
| 4 | CY016515 | A/New South Wales/5/1999 | 48 | CY015764 | A/Western Australia/36/2002 |
| 5 | CY016523 | A/New South Wales/6/1999 | 49 | CY015780 | A/Western Australia/38/2003 |
| 6 | CY016531 | A/New South Wales/8/1999 | 50 | CY015772 | A/Western Australia/37/2003 |
| 7 | CY016539 | A/New South Wales/15/1999 | 51 | CY015836 | A/Western Australia/45/2003 |
| 8 | CY016547 | A/New South Wales/16/1999 | 52 | CY015844 | A/Western Australia/46/2003 |
| 9 | CY016555 | A/New South Wales/21/1999 | 53 | CY015820 | A/Western Australia/43/2003 |
| 10 | CY016627 | A/New South Wales/4/1999 | 54 | CY015812 | A/Western Australia/42/2003 |
| 11 | CY016635 | A/New South Wales/17/1999 | 55 | CY015796 | A/Western Australia/40/2003 |
| 12 | CY016651 | A/New South Wales/22/1999 | 56 | CY015868 | A/Western Australia/50/2003 |
| 13 | CY016084 | A/New South Wales/25/2000 | 57 | CY015804 | A/Western Australia/41/2003 |
| 14 | CY016092 | A/New South Wales/27/2000 | 58 | CY015788 | A/Western Australia/39/2003 |
| 15 | CY016100 | A/New South Wales/28/2000 | 59 | CY015852 | A/Western Australia/47/2003 |
| 16 | CY016108 | A/New South Wales/29/2000 | 60 | CY015860 | A/Western Australia/48/2003 |
| 17 | CY016571 | A/New South Wales/32/2000 | 61 | CY015828 | A/Western Australia/44/2003 |
| 18 | CY016268 | A/New South Wales/33/2000 | 62 | CY015948 | A/Western Australia/60/2004 |
| 19 | CY016579 | A/New South Wales/34/2000 | 63 | CY015908 | A/Western Australia/55/2004 |
| 20 | CY016587 | A/New South Wales/35/2000 | 64 | CY015876 | A/Western Australia/51/2004 |
| 21 | CY016116 | A/New South Wales/36/2000 | 65 | CY015884 | A/Western Australia/52/2004 |
| 22 | CY016707 | A/South Australia/59/2000 | 66 | CY015892 | A/Western Australia/53/2004 |
| 23 | CY016715 | A/South Australia/62/2000 | 67 | CY015932 | A/Western Australia/58/2004 |
| 24 | CY016739 | A/South Australia/66/2000 | 68 | CY015916 | A/Western Australia/56/2004 |
| 25 | CY016747 | A/South Australia/67/2000 | 69 | CY015924 | A/Western Australia/57/2004 |
| 26 | CY016755 | A/South Australia/68/2000 | 70 | CY015972 | A/Western Australia/63/2004 |
| 27 | CY016763 | A/South Australia/71/2000 | 71 | CY015956 | A/Western Australia/61/2004 |
| 28 | CY016771 | A/South Australia/74/2000 | 72 | CY015940 | A/Western Australia/59/2004 |
| 29 | CY016491 | A/Western Australia/10/2000 | 73 | CY015900 | A/Western Australia/54/2004 |
| 30 | CY015668 | A/Western Australia/11/2000 | 74 | CY015964 | A/Western Australia/62/2004 |
| 31 | CY016499 | A/Western Australia/12/2000 | 75 | CY015980 | A/Western Australia/64/2004 |
| 32 | CY015644 | A/Western Australia/2/2000 | 76 | CY016020 | A/Western Australia/73/2005 |
| 33 | CY015652 | A/Western Australia/3/2000 | 77 | CY016044 | A/Western Australia/78/2005 |
| 34 | CY015660 | A/Western Australia/8/2000 | 78 | CY016659 | A/South Australia/23/2005 |
| 35 | CY015676 | A/Western Australia/13/2001 | 79 | CY016595 | A/South Australia/18/2005 |
| 36 | CY015692 | A/Western Australia/16/2001 | 80 | CY016603 | A/South Australia/20/2005 |
| 37 | CY013216 | A/Western Australia/15/2001 | 81 | CY015988 | A/Western Australia/65/2005 |
| 38 | CY015684 | A/Western Australia/14/2001 | 82 | CY015996 | A/Western Australia/66/2005 |
| 39 | CY015700 | A/Western Australia/17/2001 | 83 | CY016004 | A/Western Australia/68/2005 |
| 40 | CY013224 | A/Western Australia/23/2002 | 84 | CY016979 | A/Western Australia/69/2005 |
| 41 | CY015716 | A/Western Australia/26/2002 | 85 | CY016012 | A/Western Australia/70/2005 |
| 42 | CY015708 | A/Western Australia/25/2002 | 86 | CY016987 | A/Western Australia/72/2005 |
| 43 | CY015724 | A/Western Australia/28/2002 | 87 | CY016036 | A/Western Australia/75/2005 |
| 44 | CY015732 | A/Western Australia/29/2002 | 88 | CY016028 | A/Western Australia/74/2005 |

**C. 148 whole-genome sequences of H3N2 influenza A viruses from New Zealand, Australia, New York State, and globally (51 NZ, 45 AUS, 52 NY) (a representative sub-sample of 399 New Zealand sequences, 88 Australia sequences, and 413 New York State sequences) (Accession numbers correspond to PB2 gene).**

| *51 New Zealand sequences* | | | | | |
| --- | --- | --- | --- | --- | --- |
|  | Accession | Isolate |  | Accession | Isolate |
| 1 | CY007594 | A/Canterbury/01/2002 | 27 | CY012647 | A/Dunedin/3/2002 |
| 2 | CY007802 | A/Canterbury/01/2005 | 28 | CY009131 | A/Nelson Marlborough/1/2000 |
| 3 | CY007602 | A/Canterbury/02/2005 | 29 | CY011975 | A/Waikato/1/2001 |
| 4 | CY007818 | A/Canterbury/03/2005 | 30 | CY016219 | A/Waikato/1/2004 |
| 5 | CY009955 | A/Canterbury/06/2001 | 31 | CY014022 | A/Waikato/1/2005 |
| 6 | CY007466 | A/Canterbury/105/2004 | 32 | CY011655 | A/Waikato/21/2002 |
| 7 | CY007354 | A/Canterbury/12/2004 | 33 | CY012711 | A/Waikato/21/2004 |
| 8 | CY008074 | A/Canterbury/127/2005 | 34 | CY013420 | A/Waikato/36/2002 |
| 9 | CY007962 | A/Canterbury/16/2005 | 35 | CY013548 | A/Waikato/45/2004 |
| 10 | CY009123 | A/Canterbury/2/2000 | 36 | CY011967 | A/Waikato/5/2000 |
| 11 | CY007394 | A/Canterbury/20/2004 | 37 | CY013103 | A/Waikato/5/2002 |
| 12 | CY008379 | A/Canterbury/204/2005 | 38 | CY012055 | A/Waikato/58/2002 |
| 13 | CY008571 | A/Canterbury/205/2005 | 39 | CY011759 | A/Waikato/61/2003 |
| 14 | CY008643 | A/Canterbury/258/2005 | 40 | CY012639 | A/Waikato/7/2000 |
| 15 | CY009107 | A/Canterbury/3/2000 | 41 | CY013063 | A/Waikato/9/2000 |
| 16 | CY006930 | A/Canterbury/382/2003 | 42 | CY012111 | A/Wellington/1/2004 |
| 17 | CY007226 | A/Canterbury/438/2003 | 43 | CY012679 | A/Wellington/14/2004 |
| 18 | CY009155 | A/Canterbury/58/2000 | 44 | CY013476 | A/Wellington/18/2004 |
| 19 | CY009171 | A/Canterbury/61/2000 | 45 | CY012695 | A/Wellington/22/2004 |
| 20 | CY009147 | A/Canterbury/64/2000 | 46 | CY012319 | A/Wellington/34/2001 |
| 21 | CY007778 | A/Canterbury/80/2002 | 47 | CY012047 | A/Wellington/79/2002 |
| 22 | CY007786 | A/Canterbury/81/2002 | 48 | CY012687 | A/Wellington/8/2004 |
| 23 | CY008811 | A/Canterbury/85/2000 | 49 | CY014102 | A/Wellington/8/2005 |
| 24 | CY008835 | A/Canterbury/93/2000 | 50 | CY011695 | A/Wellington/86/2002 |
| 25 | CY002913 | A/Christchurch/13/2004 | 51 | CY009435 | A/West Coast/55/2001 |
| 26 | CY012623 | A/Dunedin/1/2000 |  | | |
|  | | | | | |
| *45 Australia sequences* | | | | | |
| 1 | CY016068 | A/New South Wales/13/1999 | 24 | CY015732 | A/Western Australia/29/2002 |
| 2 | CY016539 | A/New South Wales/15/1999 | 25 | CY015652 | A/Western Australia/3/2000 |
| 3 | CY016547 | A/New South Wales/16/1999 | 26 | CY015756 | A/Western Australia/35/2002 |
| 4 | CY016507 | A/New South Wales/2/1999 | 27 | CY015764 | A/Western Australia/36/2002 |
| 5 | CY016076 | A/New South Wales/20/1999 | 28 | CY015780 | A/Western Australia/38/2003 |
| 6 | CY016555 | A/New South Wales/21/1999 | 29 | CY015796 | A/Western Australia/40/2003 |
| 7 | CY016651 | A/New South Wales/22/1999 | 30 | CY015820 | A/Western Australia/43/2003 |
| 8 | CY016571 | A/New South Wales/32/2000 | 31 | CY015844 | A/Western Australia/46/2003 |
| 9 | CY016627 | A/New South Wales/4/1999 | 32 | CY015860 | A/Western Australia/48/2003 |
| 10 | CY016515 | A/New South Wales/5/1999 | 33 | CY015884 | A/Western Australia/52/2004 |
| 11 | CY016523 | A/New South Wales/6/1999 | 34 | CY015908 | A/Western Australia/55/2004 |
| 12 | CY016595 | A/South Australia/18/2005 | 35 | CY015932 | A/Western Australia/58/2004 |
| 13 | CY016715 | A/South Australia/62/2000 | 36 | CY015948 | A/Western Australia/60/2004 |
| 14 | CY016763 | A/South Australia/71/2000 | 37 | CY015956 | A/Western Australia/61/2004 |
| 15 | CY015676 | A/Western Australia/13/2001 | 38 | CY015964 | A/Western Australia/62/2004 |
| 16 | CY015684 | A/Western Australia/14/2001 | 39 | CY015972 | A/Western Australia/63/2004 |
| 17 | CY013216 | A/Western Australia/15/2001 | 40 | CY015980 | A/Western Australia/64/2004 |
| 18 | CY015692 | A/Western Australia/16/2001 | 41 | CY015988 | A/Western Australia/65/2005 |
| 19 | CY015700 | A/Western Australia/17/2001 | 42 | CY016979 | A/Western Australia/69/2005 |
| 20 | CY015644 | A/Western Australia/2/2000 | 43 | CY016987 | A/Western Australia/72/2005 |
| 21 | CY013224 | A/Western Australia/23/2002 | 44 | CY016020 | A/Western Australia/73/2005 |
| 22 | CY015708 | A/Western Australia/25/2002 | 45 | CY016028 | A/Western Australia/74/2005 |
| 23 | CY015724 | A/Western Australia/28/2002 |  | | |
|  | | | | | |
| *52 New York State sequences* | | | | | |
| 1 | CY000768 | A/New York/10/2004 | 27 | CY001631 | A/New York/267/2003 |
| 2 | CY000768 | A/New York/11/2003 | 28 | CY001639 | A/New York/268/2003 |
| 3 | CY000768 | A/New York/12/2003 | 29 | CY001647 | A/New York/269/2003 |
| 4 | CY000312 | A/New York/124/2001 | 30 | CY001663 | A/New York/284/1999 |
| 5 | CY001127 | A/New York/137/1999 | 31 | CY000032 | A/New York/32/2003 |
| 6 | CY000808 | A/New York/141/1999 | 32 | CY001887 | A/New York/327/1999 |
| 7 | CY001388 | A/New York/145/1999 | 33 | CY001999 | A/New York/332/1999 |
| 8 | CY000696 | A/New York/173/2000 | 34 | CY001903 | A/New York/333/1999 |
| 9 | CY000728 | A/New York/177/1999 | 35 | CY001919 | A/New York/336/1999 |
| 10 | CY000856 | A/New York/178/2000 | 36 | CY001927 | A/New York/337/1999 |
| 11 | CY000744 | A/New York/180/2000 | 37 | CY002583 | A/New York/339/1999 |
| 12 | CY001284 | A/New York/182/2000 | 38 | CY001943 | A/New York/340/1999 |
| 13 | CY001372 | A/New York/187/2000 | 39 | CY002447 | A/New York/365/2004 |
| 14 | CY000872 | A/New York/193/2003 | 40 | CY002039 | A/New York/382/2005 |
| 15 | CY001468 | A/New York/195/2003 | 41 | CY003132 | A/New York/406/2002 |
| 16 | CY001543 | A/New York/196/2003 | 42 | CY003167 | A/New York/412/2002 |
| 17 | CY001551 | A/New York/197/2003 | 43 | CY003207 | A/New York/418/2002 |
| 18 | CY001020 | A/New York/198/2003 | 44 | CY003800 | A/New York/423/1999 |
| 19 | CY001412 | A/New York/213/2003 | 45 | CY003808 | A/New York/428/1999 |
| 20 | CY001559 | A/New York/215/2003 | 46 | CY006075 | A/New York/455/1999 |
| 21 | CY001567 | A/New York/216/2003 | 47 | CY003687 | A/New York/485/2003 |
| 22 | CY001575 | A/New York/224/1998 | 48 | CY000264 | A/New York/52/2004 |
| 23 | CY003423 | A/New York/237/2004 | 49 | CY000964 | A/New York/59/2003 |
| 24 | CY001511 | A/New York/247/1998 | 50 | CY001436 | A/New York/76/2002 |
| 25 | CY001500 | A/New York/250/1998 | 51 | CY000288 | A/New York/86/2002 |
| 26 | CY001623 | A/New York/265/1999 | 52 | CY000304 | A/New York/96/2002 |

**D. 13 supplementary HA sequences sampled globally 1998-2005.**

| *13 Global sequences* | | | | | |
| --- | --- | --- | --- | --- | --- |
| 1 | AY531046 | A/Denmark/10/03 | 8 | DQ086160 | A/Moscow/328/2003 |
| 2 | AY531056 | A/Denmark/13/03 | 9 | DQ089637 | A/Moscow/343/2003 |
| 3 | AY531049 | A/Denmark/61/03 | 10 | AB019355 | A/Nagasaki/76/98 |
| 4 | DQ227423 | A/Fujian/411/02-like | 11 | DQ059385 | A/Oklahoma/323/03 |
| 5 | AY035589 | A/Hong Kong/1144/99 | 12 | DQ249262 | A/Taiwan/31001/2004 |
| 6 | CY002104 | A/Memphis/31/03 | 13 | DQ249259 | A/Taiwan/3640/2003 |
| 7 | CY002112 | A/Memphis/59/99 |  |  |  |

**E. 22 supplementary NA sequences sampled globally 1998-2005.**

| *22 Global sequences* | | | | | |
| --- | --- | --- | --- | --- | --- |
| 1 | DQ085799 | A/Canada/33312/99 | 12 | AY589674 | A/Kyongbuk/320/2002 |
| 2 | n/a | A/Charlottesville/03/2004 | 13 | AJ457963 | A/Latvia/1097/2000 |
| 3 | AY531025 | A/Denmark/19-2/03 | 14 | AJ457956 | A/Lyon/1242/2000 |
| 4 | AY531015 | A/Denmark/41/2000 | 15 | CY002106 | A/Memphis/31/03 |
| 5 | AJ457965 | A/Finland/620/99 | 16 | DQ090706 | A/Moscow/328/2003 |
| 6 | AF316809 | A/Greece/132/99 | 17 | AY589676 | A/Pusan/504/2002 |
| 7 | AF382329 | A/Hong Kong/1143/99 | 18 | AF533745 | A/Salta/V793/98 |
| 8 | AJ457933 | A/Hong Kong/1789/2000 | 19 | DQ249257 | A/Taiwan/31001/2004 |
| 9 | AJ307620 | A/human/Montreal/MTL327/00 | 20 | DQ249255 | A/Taiwan/4183/2004 |
| 10 | AJ307609 | A/human/Montreal/MTL71046/00 | 21 | AY947477 | A/Texas/131/2002 |
| 11 | AJ307605 | A/human/Montreal/MTL8/00 | 22 | AJ457960 | A/Valladolid/4/2001 |
